# A comparison between adsorption and photocatalytic degradation for the management of sulfamethoxazole in water

**DOI:** 10.1038/s41598-025-95947-2

**Published:** 2025-04-19

**Authors:** Ahmed Salah Elkomy, Mohamed Sh. Abdel-wahab, Nabila Shehata

**Affiliations:** 1 Materials Science and Nanotechnology Department, Faculty of Postgraduate Studies for Advanced Sciences, Beni-Suef University, Beni-Suef, 62511 Egypt; 2 Environmental Science and Industrial Development Department, Faculty of Postgraduate Studies for Advanced Sciences (PSAS), Beni-Suef University, Beni-Suef, 62511 Egypt

**Keywords:** Adsorption, Kinetic, Isotherm models, Photocatalytic degradation, Silver phosphate, Sulfamethoxazole, Environmental sciences, Chemistry

## Abstract

Sulfamethoxazole (SMX) is one of the majority of vital antibiotic medications and is widely employed for the treatment of bacterial infections. This pharmaceutical residue has been detected in surface water and sewage wastewater treatment plants (WWTP). Moreover, it has negative impacts on humans and ecosystems. The main aim of this work is to remediate water from SMX using two different water treatment techniques i.e. adsorption and photocatalytic degradation by using silver phosphate (Ag_3_PO_4_). The materials were characterized using structural (e.g. elemental dispersive X-ray spectroscopy (EDX), Fourier-transform infrared spectroscopy (FTIR), and X-ray diffraction (XRD), and morphological (Brunauer–Emmett–Teller (BET), and scanning electron microscopy (SEM)) analytical methods. The percentage of elimination of SMX at optimum solution pH, adsorbent dose, initial drug concentration and equilibrium time, was 95.15% by adsorption which corresponds to a maximum adsorption capacity (Q_max_) of 1299.7 mgg^−1^ and the removal percentage of SMX was 98.2% according to the photocatalytic degradation. Fritz-Schlunder model is the best to describe the adsorption of SMX onto Ag_3_PO_4_. Ag_3_PO_4_ can be efficiently recycled as an adsorbent using distilled water up to 4 cycles followed by ethanol 70% and turmeric extract. For the recycling of Ag_3_PO_4_ as a photocatalyst, 0.1 M NaOH is the best solvent followed by water, ethanol 70%.

## Introduction

In recent years, several forms of emerging pollutants have arisen in the water bodies. Pharmaceutical residues with a special focus on antibiotics and hormones from different classes have been found in surface water and wastewater treatment plants (WWTPs). The specific impact of antibiotics on bacterial resistance due to their frequent use and even environmental factors caused by human pollution deserves special attention^1–5^. The antibiotic-resistant microorganisms in natural media show a strong connection to their development in the environment. The biological therapy of the emerging pollutants is related to some issues such as toxicity, biodegradability and inhibition. Therefore, the presence of antibiotics in WWTPs can even affect the removal of organic residues. In addition, the lack of suitable methods to detect drugs and their metabolites is also an additional challenge in the treatment process^6^.

Sulphonamide (SMX) is an antibiotic commonly used to treat bacterial infections in humans such as urinary tract infections, bronchitis, prostatitis, upper and lower respiratory tract infections, kidney and urinary tract infections, genital infections, and skin and under the skin infections. Sulphonamide is an antibiotic that is frequently used for the treatment of infections with bacteria in humans, including genital infections, lower as well as upper respiratory tract infections, kidney and urinary tract infections, skin and under-the-skin infections, prostatitis, bronchitis, and urinary tract infections. Additionally, SMX works well against bacteria that are gram-positive and gram-negative. A recent study discovered that SMX concentrations have ranged from 0.318 to 16.009 µgmL^−1^ in wastewater samples. The high concentration of SMX was reported as 4 ngL^−1^ in freshwater reservoirs (Rawal Lake) and 0.049 mgL^−1^ in hospital wastewater. The highest concentrations of SMX were recorded in Thailand as high as 0.040 mgL^−1^ and in the USA at 0.019 mgL^−1^. However, long-term exposure to SMX may stimulate cellular metabolism, resulting in the production of Reactive Oxygen Species (ROS) by fish bodies. The process through which ROS are produced is related to the toxicity induced by various pollutants. Additionally, interactions between biological elements and xenobiotics may occur at the molecular level, leading to tissue damage that can be assessed using histological methods^7^.

Traditional sewage treatment processes and even the most powerful post-treatment methods, including filtration, sand, flocculation, coagulation, and flotation, are unable to completely eliminate SMX. Further research is being done to identify improved, high-capacity adsorbents to manage the SMX in water^8^. For example, the adsorption of SMX onto Ag_2_O NPS was examined and the maximum adsorption capacity (Q_max_) of 277.85 mgg^−1^ was achieved in 1.5 h at pH 4. The adsorption isotherm and kinetic results of experiments are defined by using the Langmuir model as well as Pseudo-second-order model, respectively^9^. SMX was also adsorbed onto cinnamon wood biochar (CWBC). The kinetic results are reported by Pseudo-second-order. The determined Q_max_ achieved 95.64 and 0.234 mgg^−1^ for pristine CWBC and CWBC amendment with soil. According to Hill and Toth model, the isotherm values for pristine CWBC, soil, and soil modified with CWBC were 113.44, 0.72, and 3.45 mgg^−1^, respectively^10^. Furthermore, the adsorption of SMX onto a composite of activated carbon (AC), TiO_2_ and silica xerogel showed that more than 90% of SMX was removed after 7 h of treatment with mass 1.5 gL^−1^ and optimum PH 4.6 ^11^. A by-product of lignite coal that was successfully converted into carbonised leonardite (cLND) was also investigated for SMX adsorption, Pseudo-second-order kinetics model seemed more appropriate for the adsorption system. The results showed that Q_max_ was 45.249 mgg^−1^^12^. For the adsorption of SMX, biochar 850BC which had been developed by KOH activation of corncob xylose residue was utilized. The kinetic and adsorption isotherm data were most effectively modelled by the Pseudo-second-order kinetic and Langmuir models, respectively. Up to 98.52% of the SMX was removed, which corresponds to Q_max_ as high as 1429 mgg^−1^^8^.

On the other hand, the photocatalytic degradation of SMX was investigated. For example, different ratios of TiO_2_ were used in a simple hydrothermal and calcination process to develop TiO_2_-CuCo_2_O_4_ heterostructures. Compared to pure CuCo_2_O_4_, TiO_2_@CuCo_2_O_4_ showed higher efficiency for SMX degradation owing to the synergistic impact between TiO_2_ and CuCo_2_O_4_. The technique produces migration of photo-generated electron-hole pairs with excellent interface contact and strong interaction between CuCo_2_O_4_ and TiO_2_. Particularly, the heterostructures have the highest charge mobility and the lowest charge transfer resistance. Since HO·, SO_4_·, and O_2_· are the highest reactive species involved in the peroxymonosulfate (PMS)-aided SMX degradation, this increases the number of photo-induced carriers that may engage in redox reactions per unit time. This enhances the photocatalytic activity and leads to the development of an n-type heterojunction^13^. Silver niobate (AgNbO_3_) was developed by a solid-state reaction. The suggested photocatalytic removal of SMX by AgNbO_3_ was found to be more effective and to proceed more quickly when the persulfate (PS) oxidant was used. The ideal conditions for the highest photocatalytic activity were 0.5 g L^−1^ AgNbO_3_, 1 mM PS, and pH 5, which resulted in 98% SMX degrading after being under visible light for 8 h. The predominant reactive species in the photodecomposition process were discovered to be photo-generated holes besides O_2_ radicals, with modest contributions from other radicals including SO_4_^14^. At the same TiO_2_ dose and photocatalytic degradation period, 97% of SMX was degraded at an initial concentration of 5 mgL^−1^ within 360 min, which was reduced to 80% for an initial concentration of 80 mgL^−1^ of SMX. At greater concentrations, a decrease in response rate was noted. For the degradation of SMX, 0.7 gL^−1^ of TiO_2_ was the ideal concentration^15^. Pd nanoparticles added on BiVO_4_ pine architecture for SMX photocatalytic degradation. The results showed that SMX was removed by Pd-BiVO_4_ and pure BiVO_4_ when exposed to visible light. Pd-BiVO_4_ effectively destroyed 98.8% of SMX after 210 min of radiation, however pure BiVO_4_ could only break down 36.3% of SMX. The active species record test showed that the photocatalytic degradation of SMX was mainly caused by h^+^ and ^·^O_2_^−^ radicals^16^.

This research aims to differentiate between two common water treatment routes; adsorption and photocatalytic degradation to manage SMX in water. Optimization of both processes in terms of SMX initial concentration, pH, adsorbent dose, and equilibrium time has been recorded. The adsorption isotherm modelling and kinetics were also studied in detail. Finally, a detailed reusability study and cost analysis have been conducted.

## Material and method

### Materials

**Fig. 1:**
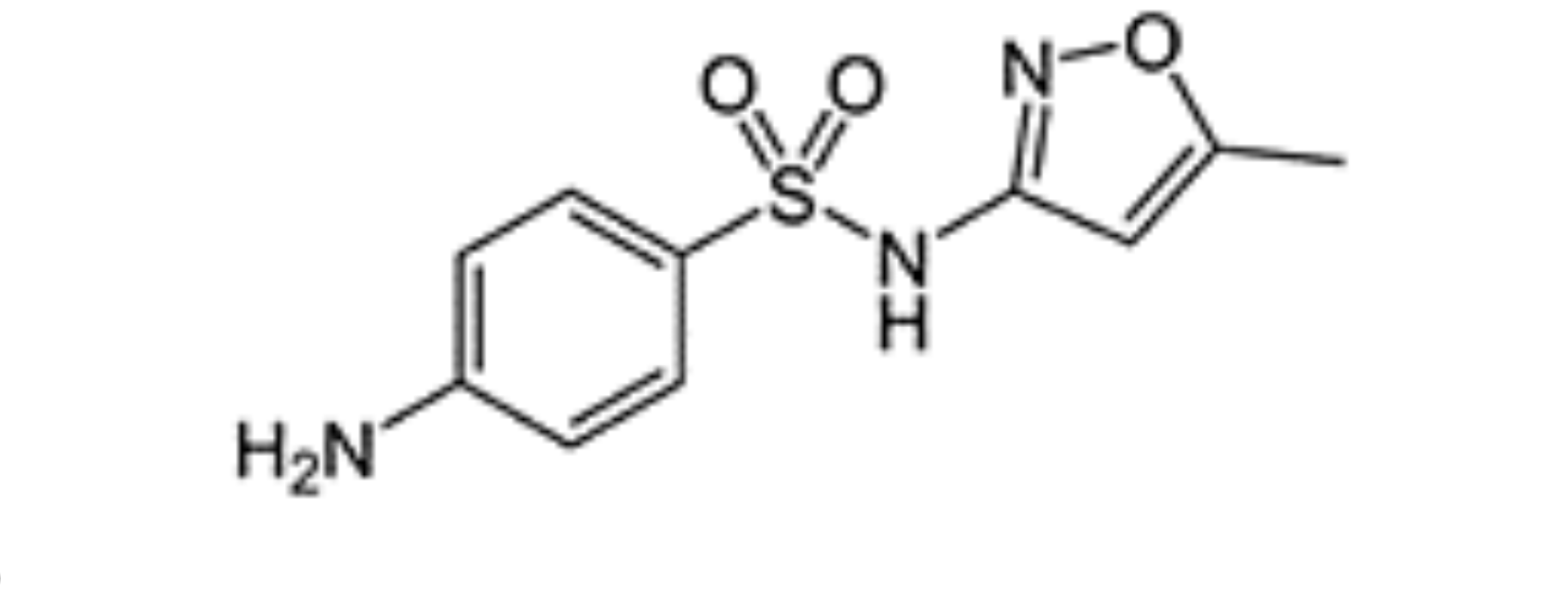
The chemical structure of SMX.

Silver nitrate was supplied from Sigma-Aldrich with 99.99% purity. Sulfamethoxazole (SMX, C_10_H_11_N_3_O_3_S; Assay 99.11%) was purchased from Anant Pharmaceuticals Pvt. Ltd., India. Potassium dihydrogen phosphate (99%) was obtained from Sigma–Aldrich. Sodium hydroxide and ethanol with a purity of 99.8% were obtained from Sigma–Aldrich and hydrochloric acid from ACS BASIC Scharlau was used. Sulphonamide antibiotic or 4-amino-N-(5methyle-1, 2-oxazol-3-yl) benzenesulfonamide has a chemical structure C_10_H_11_N_3_O_3_S as shown in Fig. 1. The industrial grade SMX was used directly without further purification.

### Methods

The synthesis of Ag_3_PO_4_ has been occurred by first, a 20 mL solution of silver nitrate in water was developed, with a concentration of 1.2 M. Then, potassium dihydrogen phosphate (KH_2_PO_4_) aqueous solution (20 ml -0.4 M) was drop-wisely added with stirring. The final mixture was continuously stirred for 60 min. After filtering, the final product has been dried at a temperature of 100 °C in a closed oven. Then, the product is washed many times with deionized water to eliminate undesirable residues^17^. Due to the low solubility of SMX in distilled water, (200 ppm) stock solution was prepared using ethanol as a solvent at room temperature.

### Characterization

Different analyses were used to evaluate Ag_3_PO_4_ both before and after adsorption and photocatalytic degradation. On a Bruker optics (Vertex 70 FT-IR, Germany) spectrometer, the FTIR spectra were recorded between 4000 and 400 cm^−1^ using the KBR pellet method. The XRD patterns are conducted using a PAN analytical (Empyrean, United Kingdom). Through the use of a field emission scanning electron microscope (FE-SEM, Zeiss, Sigma 500 VP, Germany), the surface morphology of the produced Ag_3_PO_4_ was examined. Nitrogen adsorption/desorption isotherm was used to calculate the pore size and specific surface area of Ag_3_PO_4_ according to Barreti-Joynen-Halonda (BJH) Braeuer-Emmett-Teller (BET), respectively by using TriStar II 3020 (Micromeritics, USA). The pH instrument (A∂wa–AD1030, Romania) was used to determine the pH of the solution.

### Adsorption arrays

Ag_3_PO_4_ and 20 mgL^−1^ SMX are mixed and rapidly stirred on a shaker at room temperature for 6 h at 200 rpm to prepare all group examinations. The changes were made to different adsorption conditions. These variables include changing the time intervals (5 min to 24 h), the adsorbent amount (0.01–0.1 gmL^−1^), the initial SMX concentration (10–300 mgL^−1^), and the solution pH (3–9) using 0.1 M HCL and 0.1 M NaOH. After collecting the adsorbent, the concentration of the supernatant was then measured. Following the adsorbent centrifugation to separate it, the quantities of SMX were determined with an Ultraviolet-Visible spectrophotometer (Shimadzu UV3600, Japan) at 258 nm. At equilibrium, the quantity of SMX is determined (mgg^−1^).

### Photocatalytic arrays

**Fig. 2:**
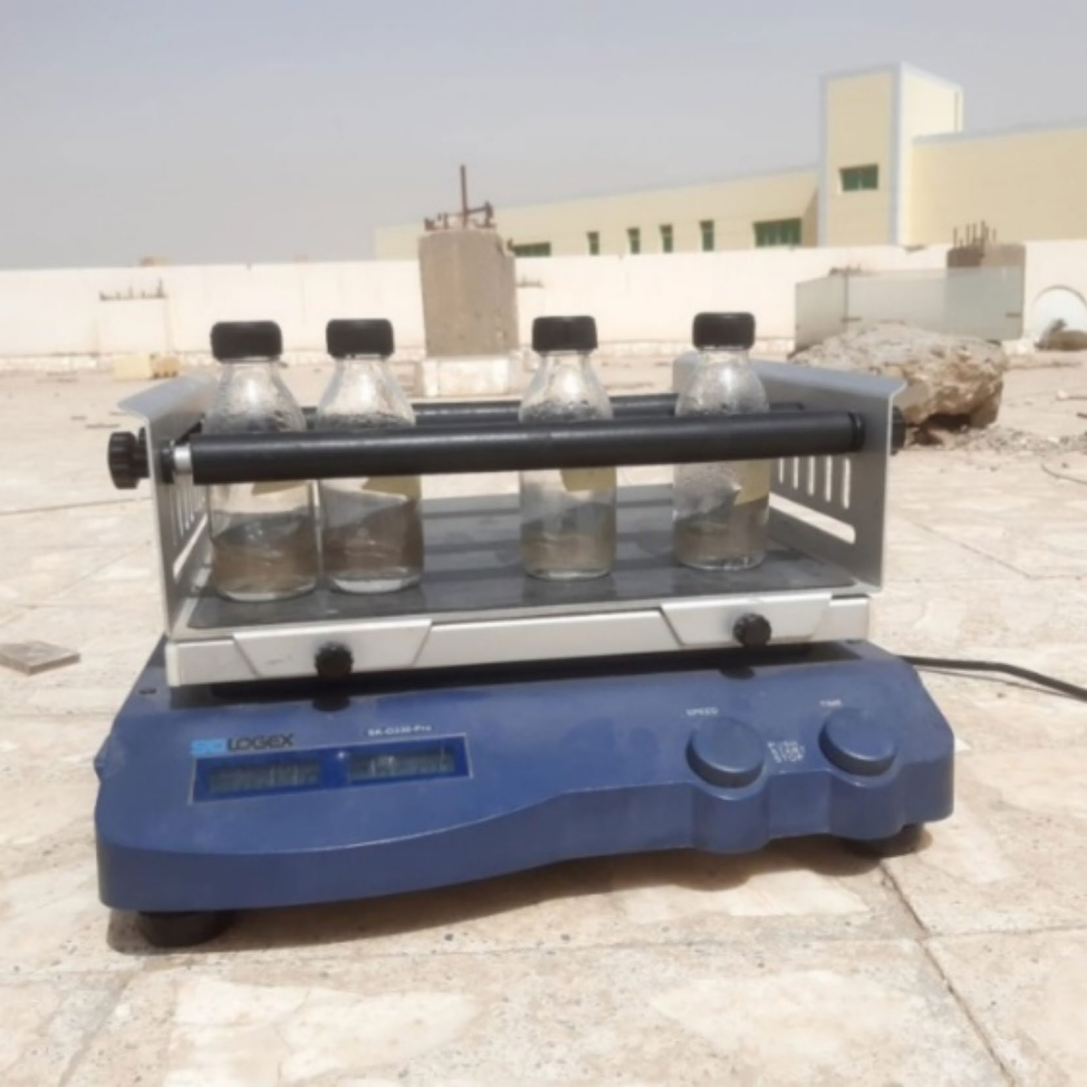
Experimental setup of the photocatalytic degradation of SMX under solar irradiation.

The photocatalytic activity of Ag_3_PO_4_ was estimated by measuring the photocatalytic degradation of SMX solutions under sun irradiation at 200 rpm on an orbital shaker (Fig. 2). The experiment was carried out in a sunny natural environment average intensity of 5.79 kW.m^− 2^ (Solar intensity between 11:00 a.m. to 4:00 p.m.) and the solute intensity index was for photocatalytic degradation tests, 0.01 g of Ag_3_PO_4_ was combined with 20 mL of 20 mgL^−1^ SMX aqueous solution.

### Reusability

In this work, the desorption of SMX from the Ag_3_PO_4_ was evaluated by different chemical solutions (H_2_O, 0.1 M NaOH, Ethanol, Acetone, 0.1 M NaOH, 20% ethanol and, 0.1 M NaOH 50% ethanol) and natural one (green tea and curcumin). Silver phosphate can be dissolved in 30 mgL^−1^ of SMX solution at pH 5. Following the adsorption experiment, the waste was collected, cleaned many times, and then dissolved for another SMX (pH 5, 30 mgL^−1^). The reusability of Ag_3_PO_4_ was examined by repeating this process three times. The following mass balance equation was used to calculate the adsorption capacity of SMX at equilibrium (mgg^−1^)

1 $${{\text{Q}}_{\text{e}}}={\text{ }}\left( {{{\text{C}}_{\text{o}}} - {{\text{C}}_{\text{e}}}} \right)*{\text{V}}/{\text{m}}$$

Where the initial as well as equilibrium concentrations for SMX are represented by the values C_0_ and C_e_ (mgL^−1^), respectively, and m is the amount of adsorbent and V is the solution volume (L). The removal efficiency of SMX (R %), was calculated according to Eq. 2.

2 $${\text{R}}\% {\text{ }}={\text{ }}\left( {{{\text{C}}_{\text{o}}} - {{\text{C}}_{\text{e}}}} \right)*{\text{1}}00/{{\text{C}}_{\text{o}}}$$

The equilibrium time is essential for determining a relation between the adsorption and the diffusion rate of adsorbate from an aqueous solution to the solid phase interface under different processes. The following formula is used to determine the quantity of SMX absorbed onto the surface of the adsorbent at time t, (q_t_).

3 $${{\text{Q}}_{\text{t}}}={\text{ }}\left( {{{\text{C}}_{\text{o}}} - {{\text{C}}_{\text{t}}}} \right)*{\text{V}}/{\text{m}}$$

C_t_ (mgL^−1^) indicate the liquid-phase adsorbate concentration at time t^18^.

### Kinetic dynamics models

The kinetics of the adsorption and photocatalytic degradation of SMX onto Ag_3_PO_4_ have been studied using five models. Pseudo-first-order (PFO) Model (Eq. 4) is based on the concept that the rate of SMX adsorption over time is directly related to the difference between the amount of SMS adsorbed over time and the amount of SMX adsorbed at equilibrium^19^. Pseudo-second-order (PSO) model is influenced by the number of sorbate ions and sites of adsorption in the liquid phase. The PSO kinetic model’s nonlinear form is given by Eq. (5). The intra-particle diffusion model (Eq. 6), describes the transfer of the solute to the surface of the sorbent particle then the transfer of the solute to the intra-particle active sites, and solute holding on these sites through sorption process. The favoured adsorption of sorbate in the micropores with greater pore width within the sorbent is the cause of the adsorption governed by the intraparticle model^20^.

4 $$\frac{{d{q_t}}}{{dt}}={k_1}\left( {{q_e} - qt} \right)$$

5 $${q_t}=\frac{{q_{e}^{2}{k_2}t}}{{{q_e}{k_2}t+1}}$$

6 $${k_i}={q_t}/{t^{\frac{1}{2}}}$$

K_1_ is the rate constant of PFO (min^−1^), k_2_ (g/mg min) is the PSO equation constant rate^21^ and is the intraparticle diffusion rate constant.

When solids undergo transformations in phase, Avrami model (Eq. 7) assesses the change while keeping a constant temperature^22^. The mixed first and second order (MFSO) models is expressed in Eq. (8)^23^.

7 $$q_t=q_e (1-e^{{{(-k_{av} t)}^{n_{av}}}})$$

8 $$q_t={q_e}\frac{1-e^{(-k_t)}}{1-f_2 e^{(-k_t)}}$$

Where n_av_ and k_av_ stand for Avrami component and the Avrami rate constant (min^−1^), respectively, *ƒ*_2_ is the dimensionless coefficient of MFSO model and K_t_ is the adsorption rate constant (g/mg min) of MFSO model.

To shed light on the photocatalytic degradation kinetics in this study, zero-order kinetic (Eq. 9) and first-order kinetic (Eq. 10) models were studied as follows^24^.

9 $${\text{C}} - {\text{Co }}={\text{ }} - {{\text{k}}_0}{\text{t}}   $$

10 $${\text{Ln}}\left( {{\text{C}}/{\text{Co}}} \right){\text{ }}={\text{ }} - {{\text{k}}_{\text{1}}}{\text{t}}$$

Where C and C_o_ are the SMX concentration (mgL^−1^) at reaction time t and time 0, respectively; ki is the first-order kinetic constant (min^−1^).

### Modelling isotherm

The adsorption isotherm modelling is defined as the relationship between the adsorption isotherm and the equilibrium concentration of an adsorbed substance in a solution as well as the quantity of adsorbed substance on the adsorbent surface. Adsorption equilibrium occurs when the phase containing the adsorbate is in communication with the adsorbent for a sufficient time after enough contact and the concentration of adsorbate throughout the solution is in dynamic equilibrium with the interface concentration^25^. The following section discusses in more detail the adsorption isotherm models under study. Langmuir model (Eq. 11) states that the same active points on the adsorbent surface are where adsorption occurs^26^. The separation factor or the dimensionless equilibrium parameter “R_L_” can be used to estimate the relationship between adsorbates and adsorbents using the basic characteristics of Langmuir isotherm parameters, as illustrated in Eq. (12). The number of the separation factor R_L_ may provide information on the type of adsorption. When R_L_ ranges from 0 to 1, adsorption is advantageous; when R_L_ ranges from more than 1, it is disadvantageous, and linear when R_L_ equals 1, adsorption is irreversible when R_L_ is equal to 0^27^. According to Freundlich model (Eq. 13), adsorption takes place on a surface that is heterogeneous with an irregular heat distribution^26^. Sips model (Eq. 14) is a combination of Freundlich and Langmuir isotherms and is a better way to define the process of adsorption on heterogeneous surfaces. The dimensionless heterogeneity factor $\:({n}_{s}$) is determines Sips isotherm equation. Sips equation is transformed into the Langmuir equation if $\:{n}_{s}$ equal 1, showing homogeneity of adsorption. The heterogeneous character of the modified adsorbent surface is confirmed by Sips isotherm constant$\:\:({n}_{s}$)^28^. This Dubinin-Radushkevich isotherm equation (Eq. 15) is used, assuming heterogeneous surface, to measure the adsorption process based on the potential theory. Temkin isotherm (Eq. 16) presupposes uniformly distributed binding energies. Redlich-Peterson isotherm model includes equations of Freundlich and Langmuir models, and the adsorption process is a hybrid one that deviates from perfect monolayer adsorption. It serves as a compromise to improve the fit of the Freundlich or Langmuir equation model. Redlich-Peterson isotherm behaves like Freundlich isotherm at large concentrations, whereas at low concentrations it approximates Henry’s law as Eq. (17). The combined characteristics of Freundlich and Langmuir isotherms in Toth isotherm model (Eq. 18). It agrees with Langmuir equation at low concentration limit and approaches Freundlich model at large concentrations. Khan isotherm (Eq. 19) is an extended model that can represent both extremes Langmuir and Freundlich types and it could be used for pure solutions. It was extended for adsorption systems with many components as well as those with a single component. For pure component adsorption isotherms, the generalized formula is expressed^29^.

11 $${q_e}=\frac{{{q_{max}}{k_l}~{c_e}}}{{1+{k_l}{c_e}}}$$

12 $${R_l}=\frac{1}{{1+{K_l}{C_0}}}$$

13 $${q_e}={k_f}{c_e}^{{\frac{1}{n}}}$$

14 $${q_e}=\frac{{{q_{{m_s}}}{k_s}C_{e}^{{{n_s}}}}}{{1+{k_s}C_{e}^{{{n_s}}}}}$$

15 $$q_e = {q_m}\:EXP({-K_{DR}(RT\:\ln(1+\frac{1}{C_e}))^2})$$

16 $${q_e}=\frac{{RT}}{{{b_T}}}\ln \left( {{A_T}{C_e}} \right)~~$$

17 $${q_e}=\frac{{{K_R}{C_e}}}{{1+{a_R}c_{e}^{\beta }}}$$

18 $${q_e}=\frac{{{q_m}{c_e}}}{{{{({K_{To}}+c_{e}^{n})}^{\frac{1}{n}}}}}$$

19 $${q_e}=\frac{{{q_m}{b_K}{C_e}}}{{{{(1+{b_K}{C_e})}^{{a_k}}}}}$$

Langmuir constant is denoted as K_L_ (L/mg). The capacity for adsorption and intensity can be expressed by K_F_ and 1/n^27^. Ks (L mg^−1^) is Sips equilibrium constant and $\:{n}_{s}$ is Sips isotherm exponent. E is the energy of adsorption (kJ/mole)^30^. Where R (KJ mol^−1^K ^−1^) is the gas constant, $\:{A}_{T}$ (L g^−1^) is Temkin equilibrium binding constant, $\:{b}_{T}$ (KJ.mol^−1^) is Temkin constant associated with sorption enthalpy, and T is the temperature (K). *β* is the exponent that ranges from 0 to 1, and K_R_ (L g^−1^) and a_R_ (L mg^−1^) are Redlich-Peterson isotherm constants^25^. n is the exponent of the Toth model which has a range (of 0 to 1) and $\:{K}_{to}$ is the model constant. It is observable that when *n* = 1, this isotherm minimizes to Langmuir equation. $\:{a}_{k}$ and $\:{b}_{k}$is Khan Model exponent and constant, respectively^30^.

## Results and discussion

### Morphological structure

**Fig. 3:**
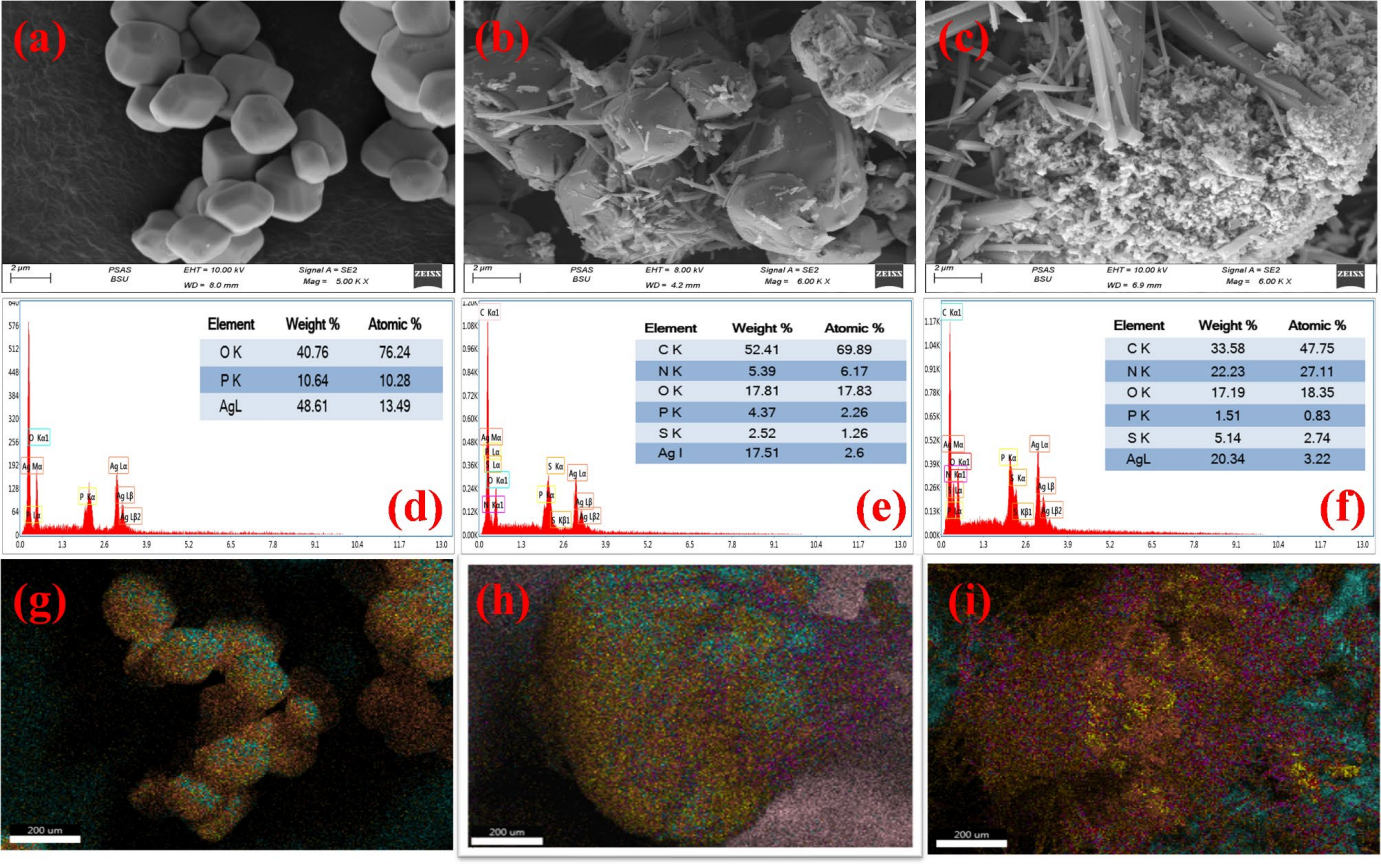
SEM images of Ag_3_PO_4_ (a), and after adsorption (b) after photocatalytic degradation (c) of SMX. EDX of Ag_3_PO_4_ (d), and after adsorption (e) and photocatalytic degradation (f) of SMX. Mapping of Ag_3_PO_4_ (g), and after adsorption (h) and photocatalytic degradation (i) of SMX.

Figure 3 illustrates the SEM images for the prepared Ag_3_PO_4_ before (Fig. 3a) and after adsorption (Fig. 3b) and photocatalytic degradation (Fig. 3c). The Ag_3_PO_4_ shape is cubic of micrometre scale in length, after the attachment of the drug onto the surface of Ag_3_PO_4_, the surface changed and became rougher and the SMX molecules were adhered to the surface of Ag_3_PO_4_ in the form of needles. The synthesized EDX spectrum of Ag_3_PO_4_ was recorded as shown in Fig. 3d. This spectrum shows the Ag, p, and O peaks alone, without any additional peaks. The absence of any impurity signals indicates that the prepared sample was pure. After adsorption (Fig. 3e) and photocatalysis (Fig. 3f), peaks for S, N and C have been appeared because of SMX. The mapping (Fig. 3g–i) shows that the element appears in all samples which informs the attachment of the SMX onto the surface of Ag_3_PO_4_.

### FT-IR results

**Fig. 4:**
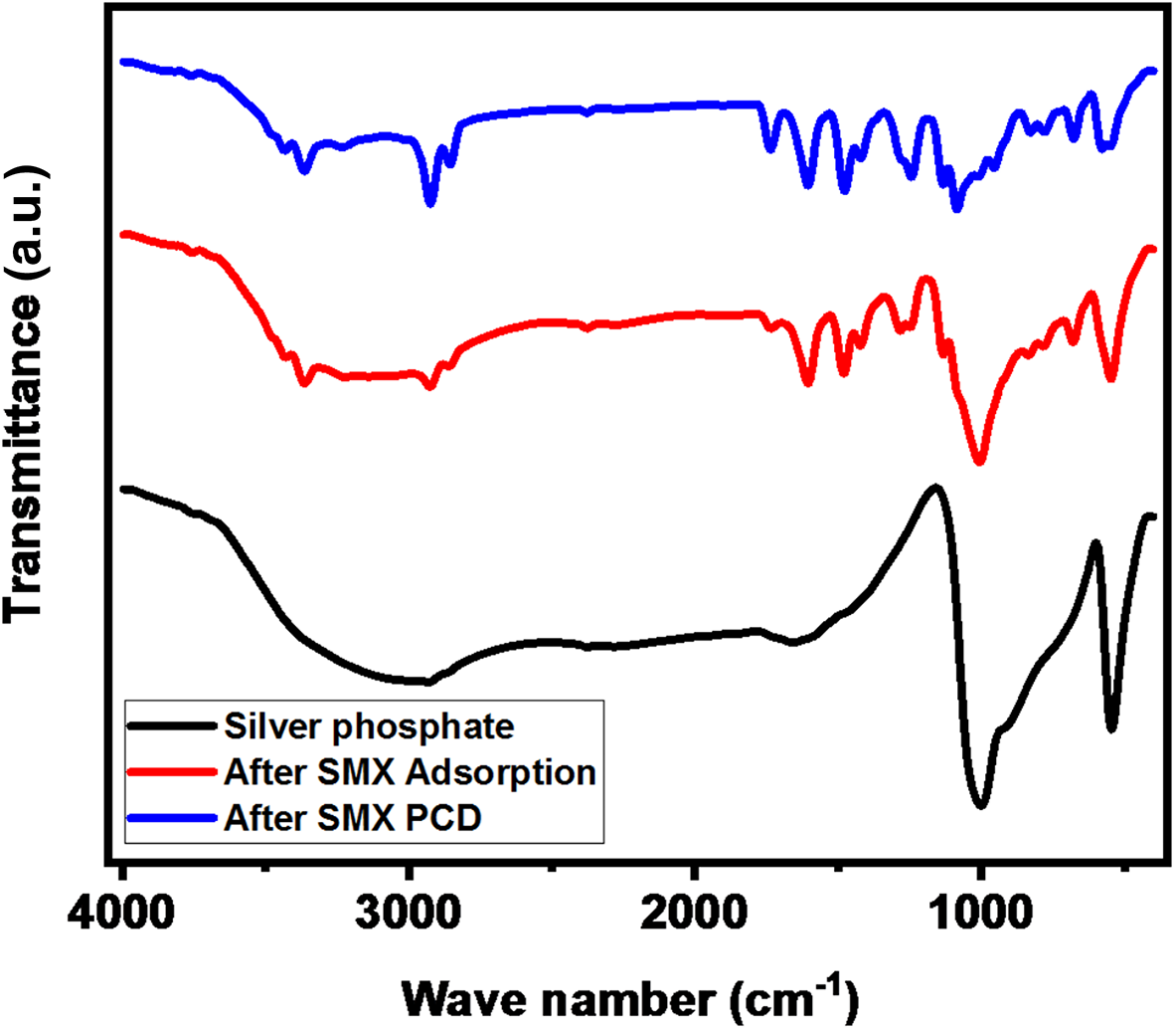
A comparison of FT-IR spectra of Ag_3_PO_4_ (a) after adsorption (b) after photocatalytic degradation (c) of SMX.

Table 1 represents the result of FT-IR where the characteristic bonds are listed along with the corresponding assignment. Following adsorption and photocatalytic degradation, the FTIR spectrum of SMX onto silver (Fig. 4, and Table 1), the FTIR spectra shows distinctive absorption bands that are associated with the silver phosphate which confirms the attachment of SMX onto the adsorbent/photocatalyst.

**Table 1:** FT-IR results of Ag_3_PO_4_, and after adsorption and photocatalytic degradation of SMX.

Bond (cm^−1^)	Ag_3_PO_4_	SMX/Ag_3_PO_4_ after PHD	SMX/Ag_3_PO_4_ after adsorption	References	Remarks
3432.682		OH stretching vibration		^31^	
			N-H	^32^	
3364.691	Stretching vibrations of O–H	Symmetric of the primary amino group (*νNH*_2_ *as*, *νNH*_2_ s)		^31^	
3232.402		Sulfonamide NH stretching		^33^	
2924.201	C–H bonds	Stretching vibration of aldehyde group C–H		^8^	
2377.020		Stretching vibrations of O–H		^31^	
1736.498		C=O extending		^31^	Distinction for SMX
1605.267		Aromatic furan moiety		^34^	Distinction for SMX
		C=C		^35^	
		Stretching vibration of C–N group		^36^	
1477.725		Sulfonamide groups (S=O)		^37^	Distinction for SMX
		CO_2_ stretching vibration		^4^	
1422.345		Bending vibration of H–O–H		^38^	
1244.815		Sulfoxide groups	(C–N)	^39^ and ^37^	Distinction for SMX
1133.699		S=O stretching		^34^	Distinction for SMX
1085.171		N–H		^40^	
955.892		O−H out-of-plane bending vibrations		^34^	
828.817		–SO_2_– bonds		^40^	Distinction for SMX
780.305		Ag_2_O		^9^	
677.851		O–S–O group vibrations and bending vibration of C= O =N group		^36^	Distinction for SMX
578.270		Molecular vibrations of phosphate (PO_4_ )^–3^		^41^	
549.556	P–O bending vibrations of PO_4_			^38^	
	Ag–O–Ag Stretching vibration			^9^	
1658.099	Stretching between phosphate and O–H group			^38^	
1000.721	Asymmetric stretching oscillations of P–O–P groups		Asymmetric stretching oscillations of P–O–P groups	^42^	
1283.422			S═O_str_	^34^	

### XRD results

**Fig. 5:**
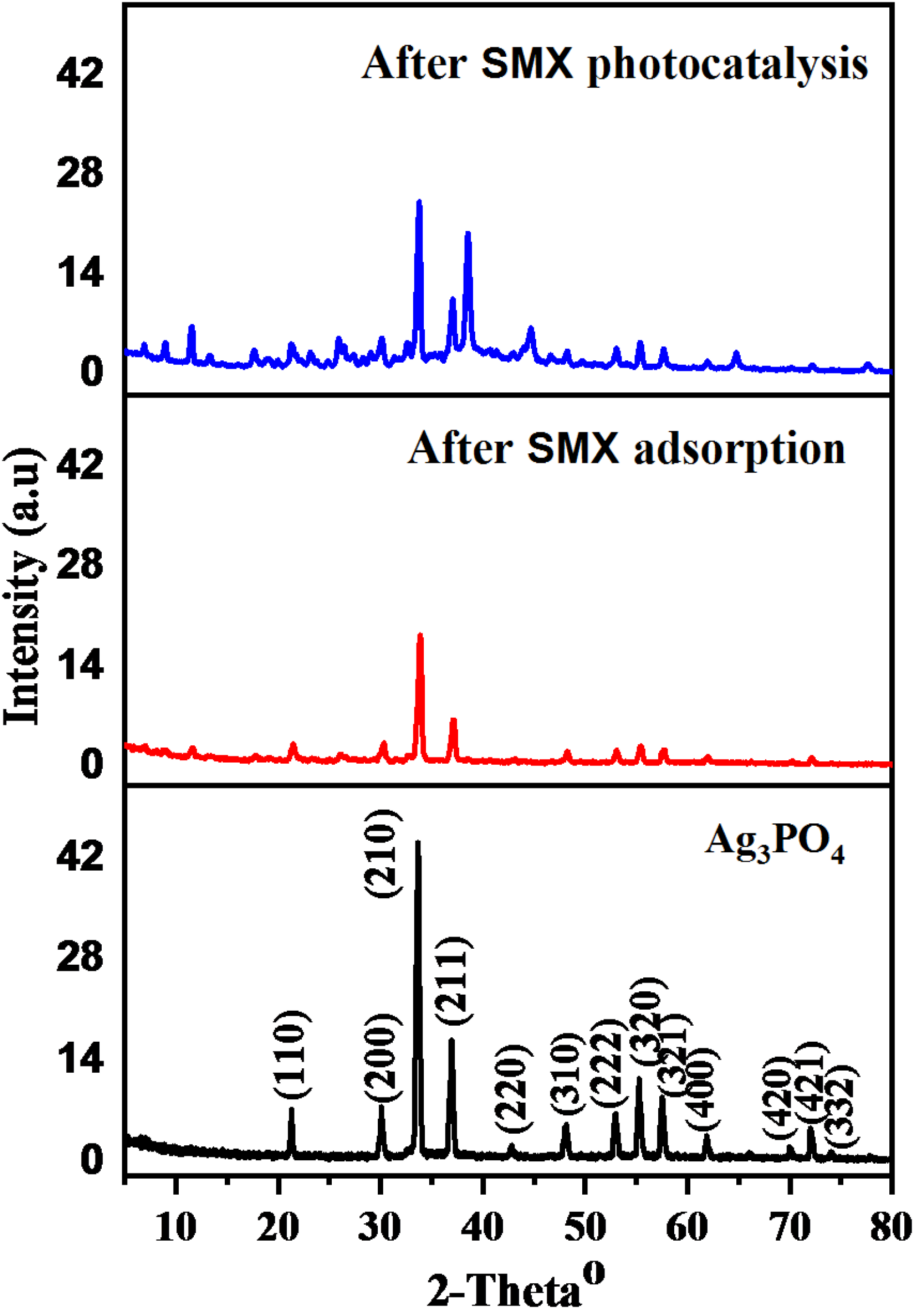
A comparison of XRD diffraction patterns of Ag_3_PO_4_ (a) and after adsorption (b) and photocatalytic degradation (c) of SMX.

X-ray diffraction analysis was used to identify the crystal structure and organization of the produced materials. As indicated in Fig. 5, the XRD pattern of Ag_3_PO_4_ matched with the standard XRD patterns of silver phosphate card 00-001-1058 and also agreed with reference card code DF 01-089-7399. It can be seen that after SMX uptake by Ag_3_PO_4_, there is a significant reduction in addition to small shifting in all the characteristic peaks of Ag_3_PO_4_ (Table 2). However, the shift in the peak locations is around 0.2° showing that no phase change on the crystalline structure of Ag_3_PO_4_ following SMX adsorption. The XRD results also showed that the Ag_3_PO_4_ pattern had new undefined peaks which may be attributed to that during the photocatalytic degradation process, Ag_3_PO_4_ undergoes severe photocorrosion, which reduced the Ag^+^ in the Ag_3_PO_4_ crystal to metallic Ag^43^ and a tiny quantity of Ag_3_PO_4_ was changed into Ag_2_O^44^, resulting in a significant change in the Ag_3_PO_4_ crystal structure. These results suggest that the adsorption process is performed physically via filling the internal surface area while the photocatalytic process occurs chemically.

**Table 2:** The changes in the diffraction patterns of Ag_3_PO_4_ before and after adsorption and photocatalytic degradation.

Ag_3_PO_4_	After adsorption of SMX	After the photocatalysis of SMX	Comments
21.3024	21.4440	21.3226	Shifting in the peaks owing to the attachment of SMX
30.0604	30.3233	30.1210	
33.6404	33.8427	33.7618	
37.0384	37.0991	37.0384	
41.9939	43.1468	42.9850	
48.1427	48.2640	48.2438	
52.9767	53.0576	53.0374	
55.2421	55.4646	55.3634	
57.5074	57.7501	57.6490	
61.8763	62.0179	61.9168	
65.9620	66.2047	66.2047	
69.9466	70.8770	70.1286	
71.9692	72.1513	72.2322	
73.9514		74.2143	
	11.5938, 17.7830 and 26.1162	6.8810, 11.5533, 13.3535, 16.7515, 17.6414, 18.9764, 20.1293, 23.1227, 24.8218, 26.4601 and 28.3209	New peaks appeared which are characteristic to SMX
	32.9123	8.9441 25.9342, 32.6291, 44.6840, 46.8482, 78.1180, 41.3264 and 64.7687	Unidentified products

### Diffuse reflectance spectra and optical band results

**Fig. 6:**
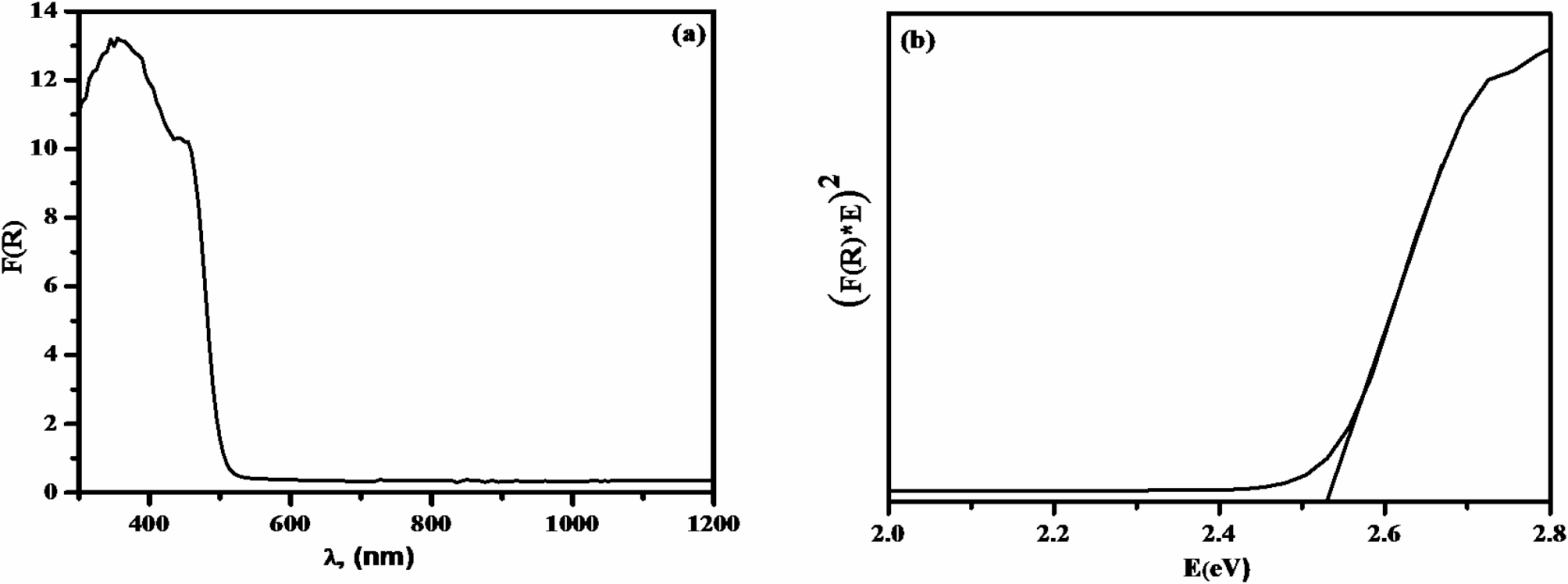
(a) Diffuse reflectance spectra, (b) Optical band gap of pure Ag_3_PO_4_.

The UV-Vis spectroscopy is being utilized to measure the absorbing characteristics of the solids (Fig. 6a and b). The spectrum of diffused reflectance is visible. The direct band gap of 2.51 eV was found to correspond to the absorption edge in pure Ag_3_PO_4_ at a wavelength of approximately 528 nm (Fig. 5a). The photocatalytic process is directly impacted by the material’s energy band gap. When a semiconductor material absorbs a photon with a band gap equal to or greater than its own, an excited electron moves from the valence band (VB) towards the conduction band (CB). The hole in the VB is caused by the electron movement from VB to CB. The extra electron-hole pair produced by the absorption of these photons leads to the dissociation of the material into free photo-holes in the valance band and photoelectrons in the conduction band. Photon-hole recombination generates energy, which promotes the photocatalytic process. For direct band gaps, the VB Eq. (20) and CB Eq. (21) have been calculated using the following formulae.

20 $${E_{VB}}={\text{ X}}\, - \,{{\text{E}}^{\text{c}}}+{\text{ }}0.{\text{5Eg}}$$

21 $${E_{CB}}={\text{ X}}\, - \,{{\text{E}}^{\text{c}}} - 0.{\text{5Eg}}$$

Where ${E_{CB}}$ is the conduction band’s energy in the equations above, and ${E_{VB}}$ is the energy of the valance band, E is the semiconductor band gap, X is the absolute electronegativity, and Ec is the energy of the free electron at the hydrogen scale. The calculated VB and CB for pure Ag_3_PO_4_ in the direct band; The measured valence band was 2.71 eV for pure Ag_3_PO_4_ and 0.20 eV for the valance band^17^.

### BET results

The distribution of pore sizes, total pore volume, and specific surface area of porous solids which are calculated using N_2_ sorption at 77 K are crucial data points in the adsorption process. The BJH was used to calculate the average pore diameter. After the photocatalysis of SMX, the specific surface area of Ag_3_PO_4_ (19.059 m^2^ g^−1^) is higher than the pristine Ag_3_PO_4_ (3.20 m^2^ g^−1^). The pore volume for Ag_3_PO_4_ is also increased to 0.0344 cm^3^ g^−1^ after photocatalytic degradation compared to the pore volume of pristine Ag_3_PO_4_ (0.005 cm^3^ g^−1^). This is in agreement with the SEM data, which show that the cubic shape of Ag_3_PO_4_ is destroyed by photocatalytic degradation, increasing cracks and curvature of the surface resulting in an increment in the specific surface area. After adsorption, the surface area and pore volume of the adsorbent were increased to 20.8524 m^2^ g^−1^ and 0.0457 cm^3^ g^−1^, respectively.

### Adsorption and photocatalysis arrays

#### pH

**Fig. 7:**
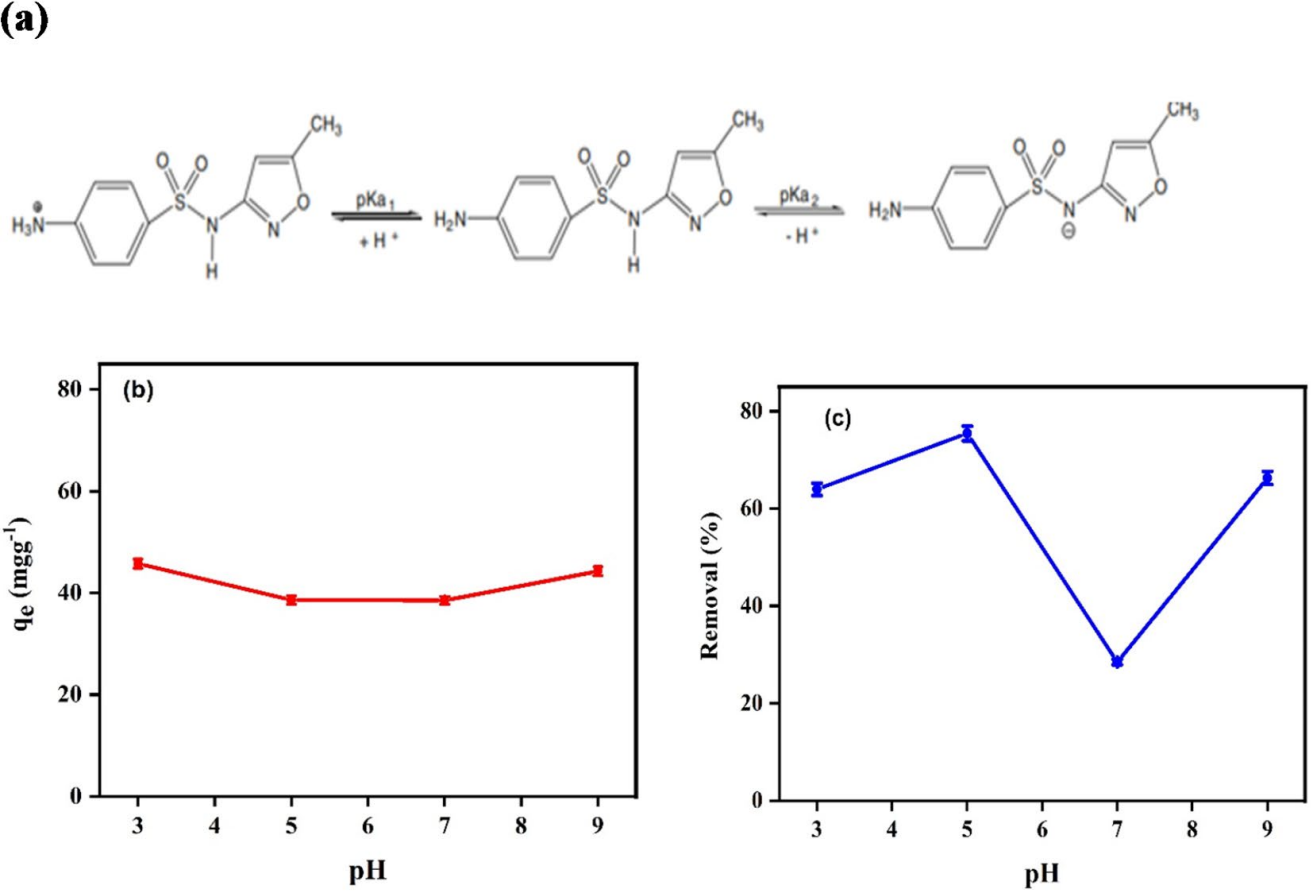
The two dissociations of SMX (a) and the effect of pH on the adsorption (b) and photocatalytic degradation (c) of SMX onto Ag_3_PO_4_ using 20 mL of SMX, adsorbent dose 0.01 g, SMX initial concentration 20 mgL^−1^ at room temperature (b).

The zeta potential of Ag_3_PO_4_ is – 29.9 mV. The results of the previously reported experiments match with the zeta potential value for Ag_3_PO_4_. The impact of pH influences not only the compound’s molecular state but also the ionisation of chemically active sites on the adsorbent. The solution pH, the drug’s property (pKa) and the adsorbent’s surface charge all have an important impact on the adsorption of ionizable pharmaceuticals; SMX possesses a basic amine group (–NH_2_) and an acidic sulphonamide group (SO_2_NH–) as shown in Fig. 7a. These variations in the functional group species of SMX at varying pH values may impact the sorption of SMX onto Ag_3_PO_4_. Hence, it has a two-step dissociation mechanism^45^. Because of its amino function group and N-heteroaromatic rings, SMX is a powerful acceptor compound. The adsorption mechanism is called the π-π electron donor acceptor. The removal of SMX was studied on the adsorbent at a pH (3–9) as shown in Fig. 7b and c. For photocatalytic degradation, the elimination effectiveness of SMX is higher under weak acid environments than under alkaline environments because of the functional groups and unsaturated bonds. This is attributed to the dissociate constant of SMX (pKa) are pKa1 = 1.85 ± 0.30 and pKa2 = 5.60 ± 0.04. It indicates that SMX occurs in neutral form when pH ranges between 1.85 and 5.6, in anionic form when pH is 5.6 or higher, and in cationic form when pH is 1.85 or lower. On the other hand, the pH_ZPC_ of Ag_3_PO_4_ is 6.7 of which mean that the surface charge becomes negative at pH above 6.7 and positive at pH s below 6.7 this explains why the adsorption capacity decrease significantly at pH 5–7 until pH = 7 followed by slowly increase in the adsorption capacity of SMX (Fig. 7b) and significantly increase in case of photocatalytic degradation the maximum removal percentage was 75.4% at PH = 5 (Fig. 7b). For adsorption it can be seen that the pH has no significant impact on the adsorption of SMX. Only small reduction in the adsorption capacity around pH 7. Moreover, the solubility of the drug plays a significant factor in the adsorption affinity of the drug toward the sorbent where at around pH 7 the solubility of the drug SMX reach its maximum value resulting in a decrease in the adsorption capacity of SMX@Ag_3_ PO_4_^46^.

#### Initial concentration

**Fig. 8:**
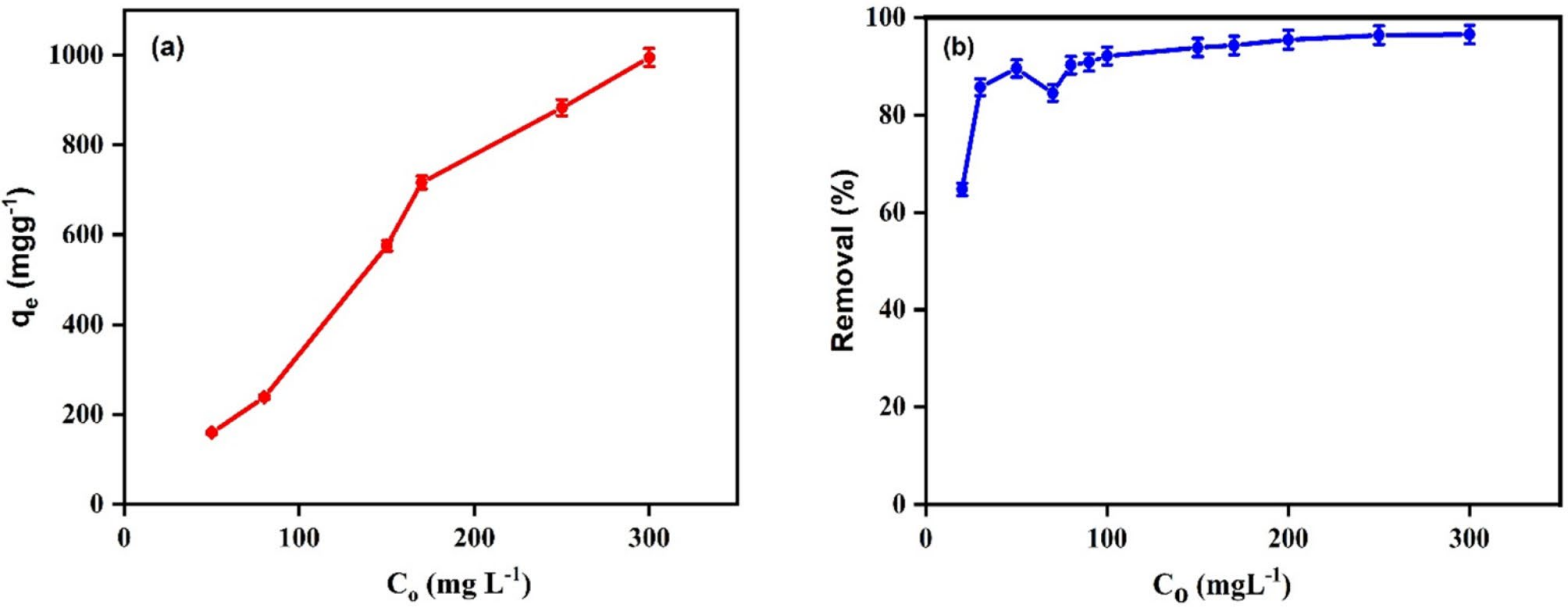
The effect of SMX initial concentration on the adsorption (a) and photocatalytic degradation (b) of SMX using Ag_3_PO_4_ for 20 mL of SMX, adsorbent dose 0.01 g and pH 5 at room temperature.

The SMX initial concentration has a significant impact on how the adsorption system functions. The relationship between (mgL^−1^) and the SMX initial concentration showed that the values of increase with the increase in the concentration of SMX. At an initial concentration above 400 mgL^−1^, there are more SMX molecules available, and more of them can compete to interact with the sorbent active sites resulting in a significant decrease in the adsorption rate, the increased from 49.9 to 1299.7 (for adsorption) as shown in Fig. 8a. For photocatalytic degradation, when the initial conc. of SMX increased from 20 to 300 mg/L the removal percentage increased from 64.7 to 96.5% and then decreased to 55.3 when the initial concentration reached to 750 as represented in Fig. 8b. However, further increase in the concentration (> 300 mg/L), no significant change has been observed. This is may be attributed to that the increase in the concentration of SMX while the quantity of the developed ^·^OH and O^·^_2_^−^ in the solution remain constant, results in a constant decomposition of SMX in the solution.

#### Dose impact

**Fig. 9:**
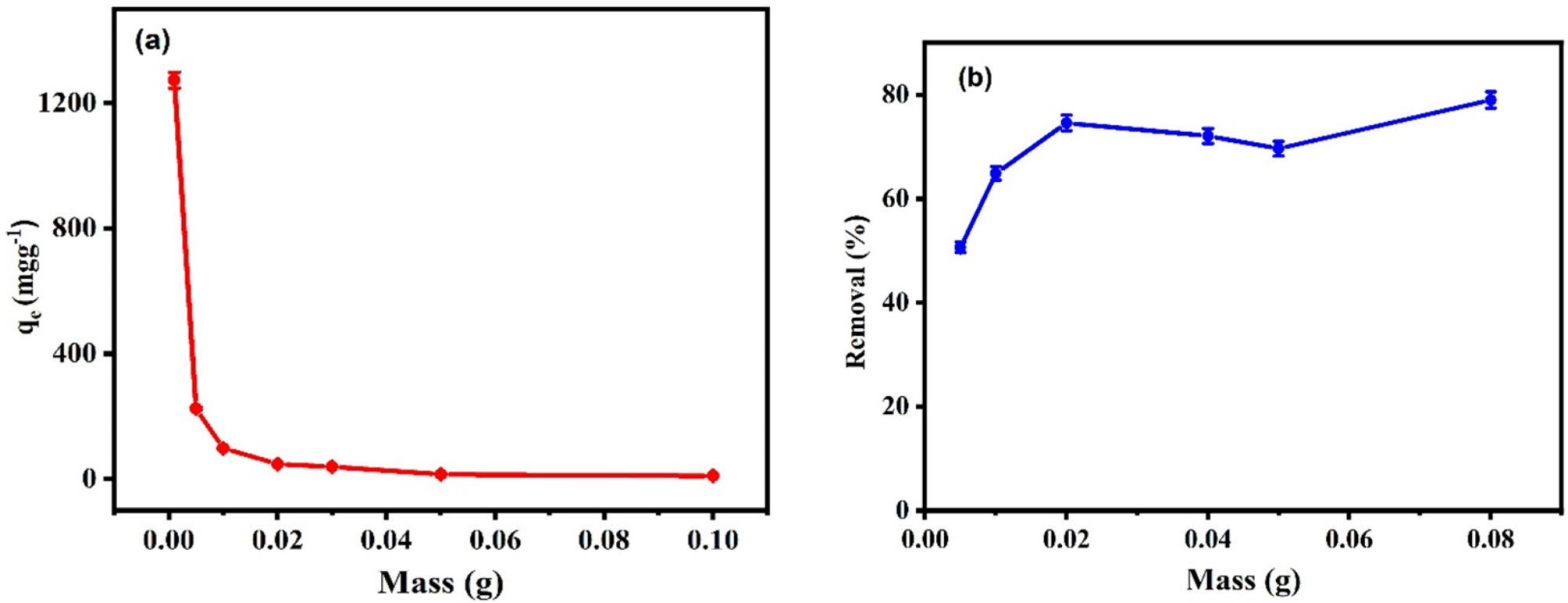
Effect of Ag_3_PO_4_ dose on SMX removal by adsorption (a) and photocatalytic degradation (b) using 20 mL SMX solution, pH 5, SMX initial concentration 20 mgL^−1^ at room temperature and mass 0.001 g to 0.1 g.

Figure 9 shows the effect of the mass of Ag_3_PO_4_ on the elimination of SMX, the results show that SMX adsorption capacity increased when the adsorbent dose was reduced, reaching its greatest level at 0.001 g. As the dose of the adsorbent increased, the amount of available active sites for adsorption decreased due to Ag_3_ PO_4_ aggregation, which reduced the amount of available active sites for adsorption/photocatalytic degradation by decreasing the specific surface area. For photocatalytic degradation, in order to prevent the recombination of catalyst electron hole pairs, the effect of the catalyst dose was examined to determine its effect on accelerating the degradation efficiency of the SMX. When the dose was increased from 0.005 to 0.02 g, the degradation percent was increased significantly from 50.6 to 74.6%, further increase in the dose up to 0.08 didn’t enhance significantly the percentage of removal. As a result, the optimum dose of the photocatalyst was 0.02 g. This could be used to illustrate that as the catalyst dose increases, sufficient surface area becomes available to serve the photo degradation process, and the available active sites on the catalyst responsible for degradation will be generated forcing water molecules to develop hydroxyl (^·^OH) and superoxide (O^·^_2_^−^) radicals, which in turn serve the degradation rate. These results are agreed with a previous work on metronidazole degradation^47,48^.

#### Contact time

**Fig. 10:**
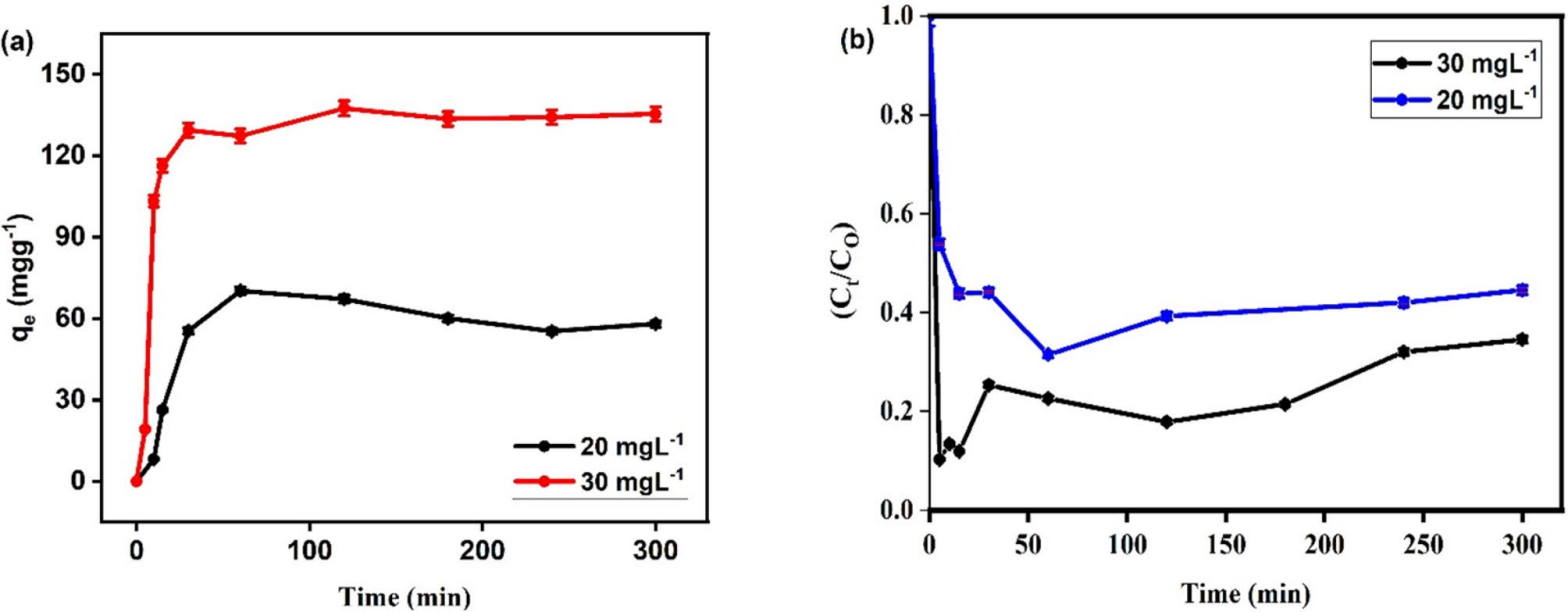
Effect of contact time SMX on the removal of SMX using adsorption (a) and photodegradation (b) by Ag_3_PO_4_ at SMA initial concentrations 20 and 30 mgL^−1^, pH 5, adsorbent dose 0.01 g at room temperature.

Figure 10 shows the adsorption of SMX by Ag_3_PO_4_. Because there are so many active sites in the material, the data show that the removal of SMX was high within the first 100 min (Fig. 10a), as the time increased from 0 to 100 min the adsorptivity increased from 0 to 137.45 (for 30 mgL^−1^) and 70.20 mgg^−1^ (for 20 mgL^−1^) followed by an equilibrium stage where no further increase was attained. For photocatalytic degradation (Fig. 10b), it increased removal of SMX was high within the first 15 min follow by an equilibrium stage where no further increase was attained. This suggests that the adsorbent is saturated and has reached its maximum capacity.

#### Adsorption isotherm studies

**Fig. 11:**
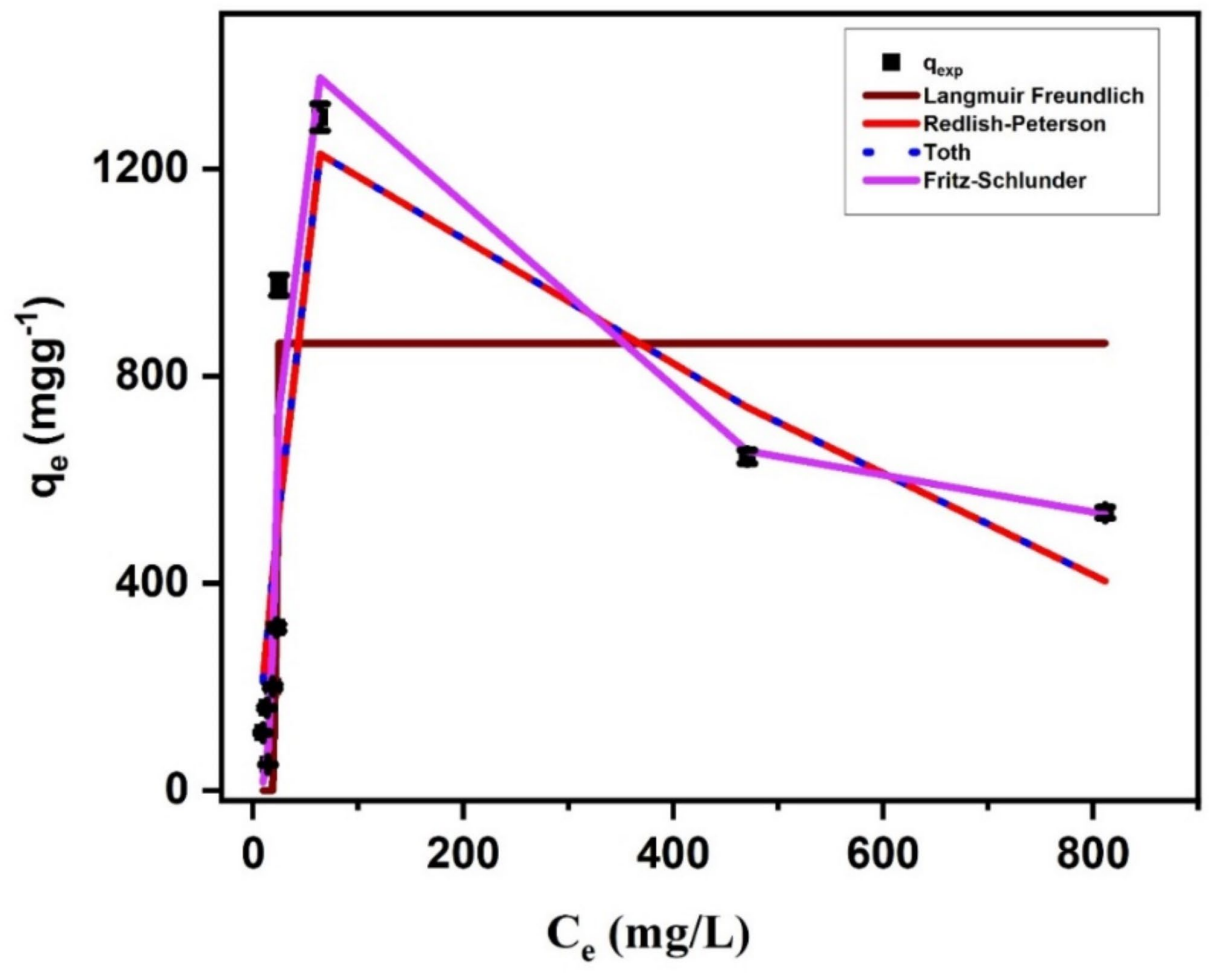
Equilibrium adsorbed amount of SMX onto Ag_3_Po_4_ versus SMX equilibrium concentration using Langmuir, Freundlich, Dubinin-Radushkevich, Temkin, Redlich-Peterson, Sips, Langmuir-Freundlich isotherm, Toth, Baudu, Kahn and Fritz schlunder models.

The adsorption isotherm modelling is used to describe the maximum adsorption of SMX onto Ag_3_PO_4_. Table 3 includes a list of all variables with regard to these isothermal models. Fritz-schlunder is the best to describe the experimental data of SMX adsorption onto Ag_3_PO_4_ with high R^2^ (0.90). Additionally, the theoretical values of adsorption quantity are close to the experimental value (Fig. 10a). Followed by Redlich-Peterson, Toth and Langmuir-Freundlich isotherm models with moderate correlation coefficients (R^2^ = 0.71–0.75). On the other hand, Freundlich (R^2^ = 0.17), Dubinin-Radushkevich (R^2^ = 0.43), Dubinin-Radushkevich (R^2^ = 0.57), Baudu (R^2^ = 0.17), sips (R^2^ = 0.2), Temkin (R^2^ = 0.23) failed to describe the adsorption system understudies since the correlation coefficients according to these models are low. Also Langmuir model is not suitable for SMX@Ag_3_PO_4_ since the calculated values are far away than the experimental one (Fig. 11).

**Table 3:** The parameters involved in the adsorption isothermal modelling of SMX onto Ag_3_PO_4_ models.

Adsorption models		Parameter	Value	Adsorption models		Parameter	Value
Two parameters isotherm	Langmuir	q_max_ [mgg^−1^]	18882.4	Three parameters isotherm	Redlich-Peterson	K_R_ [L g^−1^]	22.59
		K_L_ [L.mg^−1^]	0.001			a_R_ [Lmg^−1^]	1.77E–05
		R^2^	0.77			Β	2.2
	Freundlich	K_f_ [L.mg^−1^]	230.77			R^2^	0.75
		1/n	0.18		Sips	Q_m_ [mgg^−1^]	1300
		R^2^	0.17			Ks [L mg^−1^ ]	1.63E–07
	Dubinin-Radushkevich	q_max_ [mgg^−1^]	852.12			1/n	5.12
		K_ad_ [mol^2^/kJ^2^]	0.003			R^2^	0.2
		R^2^	0.43		Langmuir-Freundlich isotherm	*q*_MLF_ [mgg^−1^]	864.03
	Temkin	b_T_ [KJmol^−1^]	20.61			*K*_LF_	0.05
		A_T_ [L g^−1^]	1.14			*M*_LF_	129
		R^2^	0.23			R^2^	0.711
Four-parameters isotherm	Baudu	q_m_ [mgg^−1^]	230.76		Toth	K_e_	49.68
		b_0_	0.04			K_L_	1.77E–05
		x	0.18			n	2.2
		y	14.97			R^2^	0.75
		R^2^	0.17				
Five-parameters isotherm	Fritz Schlender	Q _mFSS_ [mgg^−1^]	91.84				
		K_1_	7.70E–06				
		K_2_	1.05E–07				
		m_1_	4.48				
		m_2_	4.86				
		R^2^	0.90				

#### Adsorption kinetics

Different kinetic models such as PFO, PFO, MFSO, Avarmi, and IPD models were employed to examine the kinetics of the system under study. Table 4 is a list of the kinetic parameters for the various kinetic models for the adsorption of SMX onto Ag_3_PO_4_. Fitting of the kinetic model to the experimental data is evaluated based on the values of R^2^ and the agreement between the calculated and experimental data. Additionally, the calculated adsorption capacity (q_t_) values and the experimental adsorption capacities of the four models are very similar. On the other hand, the intraparticle diffusion model is not suitable for the adsorption of SMX onto Ag_3_PO_4_ since the experiential data and calculated are not matched and R^2^ values are very low. This suggests that the film diffusion is the controlling rate rather than the pore diffusion.

**Fig. 12:**
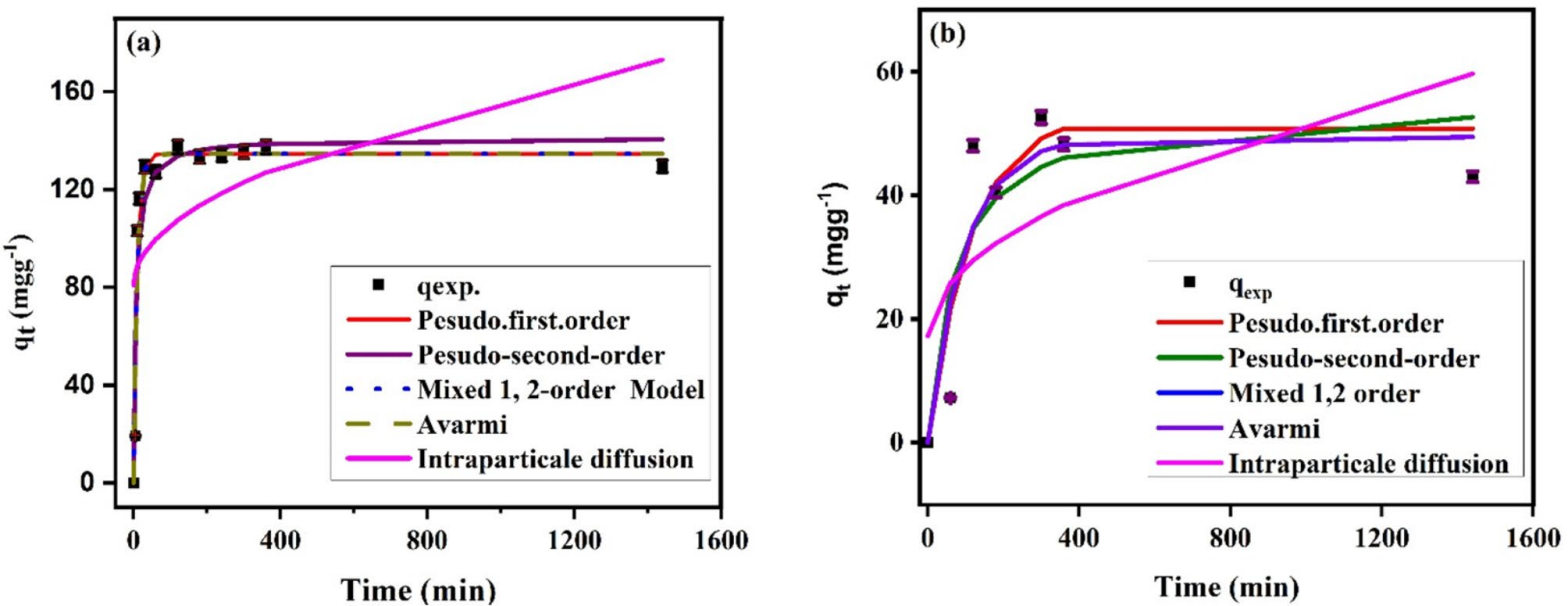
The kinetic data of SMX adsorption at initial concentrations 20 mgL^−1^ (a) and 30 (b) mgL^−1^ using Pseudo-first -order, Pseudo-second–order, mixed 1,2-order model, Avarmi and intraparticle diffusion models.

At lower concentration of SMX (20 mgL^−1^) values (Fig. 12a), the adsorption of SMX onto Ag_3_PO_4_ is described by Pseudo first order, mixed order, and Avrami with (R^2^ 0.93), and the estimated values of adsorption capacity are agreed with the experimental values (Fig. 12b). PSO could also fit the data but with lower R^2^ (0.89) compared to the other three previous models and the calculated adsorption quantity (141.22 mgg^−1^) is close to the experimental (137.4 mgg^−1^). Concerning IPD model, the calculated values according to this model are differ greatly from the results of the experiments, also the correlation coefficient is extremely low. For SMX initial concentration, 30 mgg^−1^, the models follow the same trend. The fitting of the kinetic models follows the order: PFO, MFSO and Avrami > PSO > > IPD model.

For the photocatalytic degradation of SMX by Ag_3_PO_4_, the results in Table 4 suggest that zero-degree kinetic model is more suitable at lower SMX concentration than first degree kinetic model. Where the values of R^2^ for SMX initial conc 20 mgL^−1^ (0.983) is higher than that at 30 mgL^−1^ (0.979) in case of zero-degree kinetics. On the other hands, the first-degree kinetic model represents higher R^2^ at SMX initial conc 30 mgL^−1^ (0.983) compared to SMX initial conc 20 mgL^−1^ (0.968). However, the kinetics of the photocatalytic degradation of SMX need to be investigated much more to model and implement better processes on a large scale.

In conclusion, the initial concentration of the SMX plays an important role in the kinetic modelling of the adsorption as well photocatalytic degradation of SMX by Ag_3_PO_4_.

**Table 4:** The parameters of the adsorption and photocatalytic models for SMX uptake onto Ag_3_PO_4_.

Models	Parameters	Adsorption		Models	Parameters	Photocatalytic degradation	
		Conc. (30 mgL^−1^)	Conc. (20 mgL^−1^)			Conc. (30 mgL^−1^)	Conc. (20 mgL^−1^)
PFO	q_e_ [mgg^−1^]	134.7	53.13	Zero-degree kinetics	K_0_ [min^−1^]	0.1461	0.099
	k_1_ [Lmg^−1^]	0.1	0.009		R^2^	0.979	0.983
	R^2^	0.93	0.85	First-degree kinetics	k_1_ [min^−1^]	0.0055	0.0029
PSO	q_e_ [mgg^−1^]	141.22	55.27		R^2^	0.983	0.968
	k_2_	0.001	0.0003				
	R^2^	0.89	0.76				
MFSO	q_e_ [mgg^−1^]	134.7	49.44				
	K	0.1	0.01				
	*f*_2_	0	0				
	R^2^	0.93	0.82				
Avrami	q_e_ [mgg^−1^]	134.7	49.44				
	k_av_	0.33	0.1				
	*n*_av_	0.3	0.098				
	R^2^	0.93	0.82				
IPD	k_ip_ [mgg^−1^ min^0.5^]	2.43	1.12				
	*c*_ip_ [mgg^−1^]	80.90	17.25				
	R^2^	0.29	0.39				

#### Effect of recyclability

**Fig. 13:**
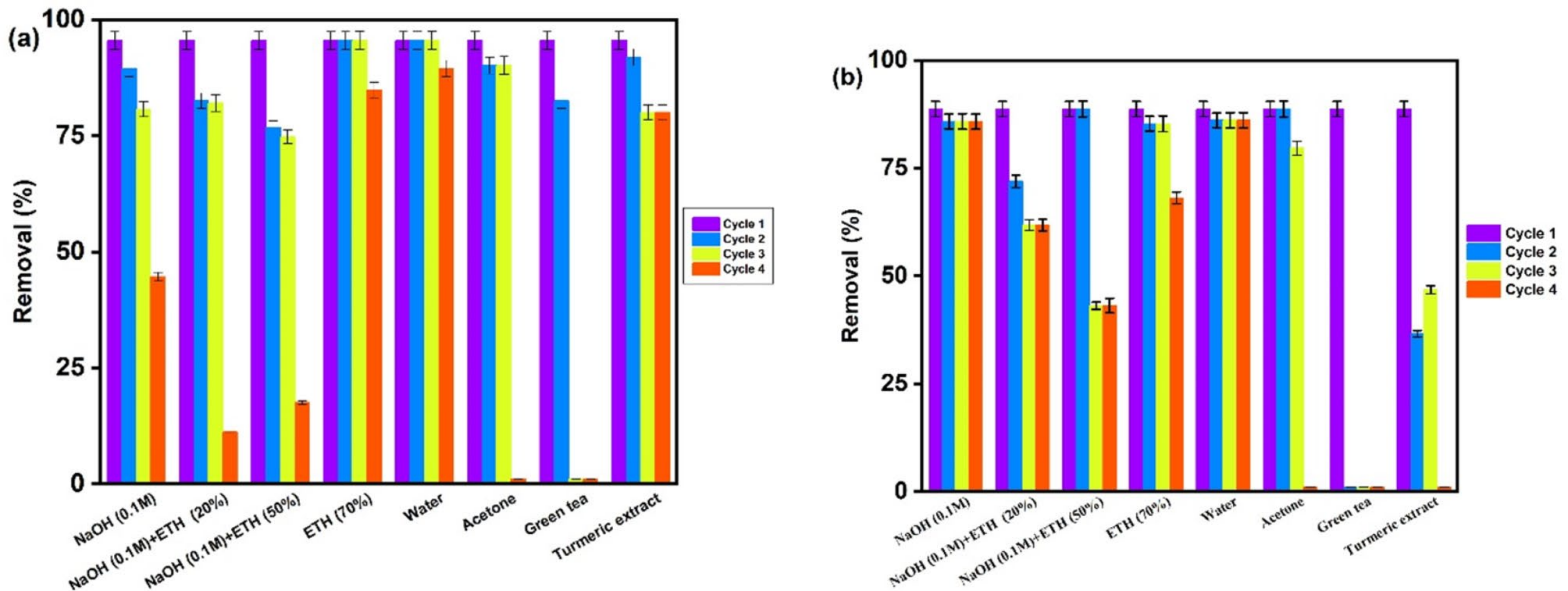
Reusability of Ag_3_PO_4_ as adsorbent (a) and as a photocatalyst (b) using different solvents/reagents (0.1 NaOH, ethanol 20% 0.1 NaOH, ethanol 50% 0.1 NaOH, ethanol 70%, water, acetone, green tea, turmeric extract).

If the adsorbent material is recycled and utilized again, the adsorption process might become cheaper to perform in addition to decreasing the negative burden on the environment. Hence, different chemical solvent have been investigated for the regeneration of the exhausted Ag_3_PO_4_ such as 0.1 M NaOH, ethanol 20% + 0.1 M NaOH, ethanol 50% + 0.1 M NaOH, ethanol 70% and acetone in addition to green solvent such as water, green tea and turmeric extract. Surprisingly, the results (Fig. 13a) show that Ag_3_PO_4_ can be efficiently recycled as an adsorbent using distilled water up to 4 cycles followed by ethanol 70% and turmeric extract. Other solvents such as 0.1 M NaOH, ethanol 20% + 0.1 M NaOH, ethanol 50% + 0.1 M NaOH recorded acceptable performance in the reusability of Ag_3_PO_4_. For the recycling of Ag_3_PO_4_ as a photocatalyst (Fig. 13b), 0.1 M NaOH is best solvent followed by water, ethanol 70%. However, turmeric extract recorded promising results in the recycling process of the exhausted sorbent.

#### Cost analysis

The cost of utilizing these adsorbents to treat wastewater should be considered, as it is one of the main points and challenges affecting the marketing of adsorption technology. Table 5 shows the cost breakdown of the production of the adsorbent/catalyst. It can be seen that the cost of one gram of Ag_3_PO_4_ is 1451.06 L.E which is high compared to commercial adsorbent. However, it can be seen that the high price is attributed to the high cost of AgNO_3_. Subsequently, future studies should focus on replacing this precursor with a low-cost one.

**Table 5:** The cost analysis of the developed Ag_3_PO_4_.

Materials	Quantity unit	Unit price (L.E.)	Quantity	Total price (L.E)
AgNO_3_	g	345	4	1380
KH_2_PO_4_	g	22	1	22
The total cost of material is 1402 L.E				

Energy	Time (h)	Power max (kw)	Unit cost (L.E.kwH)	Energy cost (L.E)
Magnetic stirrer	1	1.06	1	1.06
Dryer	24	1	2	48
The total cost of energy is 49.06 L.E				
The overall cost of 1 g of Ag_3_PO_4_ is 1451.06 L.E				

#### Recent work on SMX removal from water

Table 6 lists the recent studies on the removal of SMX using different adsorbents and catalysts along with the optimum conditions.

**Table 6:** A comparison of adsorption capacities of different materials towards SMX.

Material	Mechanism	Dose	Initial cocn.	Contact time	pH	q_max_ (mgg^–1^) or (removal %)	Reference
Microalgae exopolysaccharide/ alginate microsphere	Adsorption	30 g	10 mgL^−1^	90 min	7	142.49 (> 90%)	^49^
Activated sludge-based biochar		80 mg	100 mgL^−1^	24 h	4	45.6	^50^
Fe_3_O_4_/EXG/ Cellulose		0.05 mg	20 mgL^−1^	90 min	4	3.9	^51^
chitosan-carbon nanotube hydrogel beads		1.5 gL^−1^	40 mgL^−1^	12 h	7	25.17	^52^
Calcined layered double hydroxides		10	1-500	1 h	7	4314	^53^
N-doped zeolite beta-templated carbon		10	100	10 min	4	1367	^54^
Ag_3_PO_4_		0.01 g	30–400 mgL^−1^	100 min	5	1299.7	This study
Photocatlyst		Dose	Initial cocn.	Contact time	pH	(removal %)	Reference
TiO_2_& AC (calcined)	Photocayaltic degradation	100 mgL^−1^	100 µgL^−1^	2 h	–	95.6%.	^55^
S-scheme Fe_2_O_3_/g-C_3_N_4_		0.3 gL^−1^	10 mgL^−1^	30 min	6	99.2%	^56^
P@g-C_3_N_4_		10 mgL^−1^	–	3 h	–	70%	^57^
SrTiO_3_& electrospun carbon fibers composite		20 mg	5 mgL^−1^	5 h	7	90%	^58^
Ag_3_PO_4_		0.01 g	30–400 mgL^−1^	100 min	5	98.2%	This work

### Strengths and limitations of the study

Both adsorption and photocatalytic processes for SMX which is used as an antibiotic for treating bacterial infections in humans using Ag_3_PO_4_ as a powdered material have their unique advantages such as simplicity, and effectiveness, and can be regenerated and reused. In our work, we succeeded in removing the SMX 95.15% by adsorption which corresponds to a maximum adsorption capacity of 1299.7 mgg^−1^ and the removal percentage of SMX was 98.2% according to the photocatalytic degradation. Due to not need for light in the adsorption process, we can depend on the adsorption process instead of using the photocatalytic process or we can combine the two techniques to improve the removal efficiency. This study can be applied to the removal of different pharmaceutical drugs in water. On the other hand, the limitations of using Ag_3_PO_4_ as a powdered material for adsorption and photocatalytic processes for the SMX are the high cost of the chemicals used in the preparation such as AgNO_3_, pH sensitivity, light dependency, and temperature. One of the most important limitations that hinder the use of powder materials in the adsorption and photocatalytic on the industrial scale is that hard to separate, reuse and produce environmental risks. Another limitation of this study is the stability of Ag_3_PO_4_ as adsorbent and photocatalyst. Also, we didn’t apply the prepared Ag_3_PO_4_ on real wastewater.

## Conclusion and perspectives

In this work, the simple co-precipitation approach is utilised to synthesise Ag_3_PO_4_, which was then employed as a possible adsorbent/photocatalyst to eliminate SMX from water. In both routes; adsorption and photocatalytic degradation, the elimination of SMX from water is highly dependent on the operating conditions and it was found that the optimum conditions are; pH 5, SMX initial concentration (300 mgL^−1^), contact time 100 min, adsorbent dose 0.001 g and photocatalyst dose 0.02 g. However, the photocatalytic degradation route recorded high removal 98.2% compared to the adsorption route 95.15% (Qmax 1299.7 mgg^−1^). The high removal percentage of this antibiotic according to both routes is excellent and it is a good sign for facing the recent challenges of bacterial resistance to SMX which is considered another contaminant of emergent concern. The reusability study showed that water can be used as a green solvent to regenerate the exhausted adsorbent up to 4 cycles. Moreover, turmeric extract recorded promising results in the regeneration of Ag_3_PO_4_. Further studies should focus on minimizing the cost of the developed adsorbent/photocatalyst to be used on a large scale.

In future work, it is highly recommended to synthesis a composite with Ag_3_PO_4_ including carbon materials to improve the efficiency of removing the pharmaceutical drugs and applying the prepared materials to real wastewater. Also, we are planning to use the prepared Ag_3_PO_4_ as a thin film on a glass substrate to make the reusability easier and apply it on the industrial scale instead of using it on the lab scale. Also, it is strongly recommended to investigate the performance of Ag_3_PO_4_ in the management of SMX in real sewage.

## Data Availability

All data listed or discussed during this work are included in this published article.
